# Angiotensin-converting enzyme inhibition and food restriction restore delayed preconditioning in diabetic mice

**DOI:** 10.1186/1475-2840-12-36

**Published:** 2013-02-23

**Authors:** Gerry Van der Mieren, Ines Nevelsteen, Annelies Vanderper, Wouter Oosterlinck, Willem Flameng, Paul Herijgers

**Affiliations:** 1 Department of Cardiovascular Sciences, Research Unit Experimental Cardiac Surgery, K.U. Leuven, Herestraat 49, B-3000, Leuven, Belgium

**Keywords:** Myocardial protection, Preconditioning, Ischemia/reperfusion injury, Diabetes mellitus, Metabolic syndrome

## Abstract

**Background:**

Classical and delayed preconditioning are powerful endogenous protection mechanisms against ischemia-reperfusion damage. However, it is still uncertain whether delayed preconditioning can effectively salvage myocardium in patients with co-morbidities, such as diabetes and the metabolic syndrome. We investigated delayed preconditioning in mice models of type II diabetes and the metabolic syndrome and investigated interventions to optimize the preconditioning potential.

**Methods:**

Hypoxic preconditioning was induced in C57Bl6-mice (WT), leptin deficient ob/ob (model for type II diabetes) and double knock-out (DKO) mice with combined leptin and LDL-receptor deficiency (model for metabolic syndrome). Twenty-four hours later, 30 min of regional ischemia was followed by 60 min reperfusion. Left ventricular contractility and infarct size were studied. The effect of 12 weeks food restriction or angiotensin-converting enzyme inhibition (ACE-I) on this was investigated. Differences between groups were analyzed for statistical significance by student’s *t*-test or one-way ANOVA followed by a Fisher’s LSD post hoc test. Factorial ANOVA was used to determine the interaction term between preconditioning and treatments, followed by a Fisher’s LSD post hoc test. Two-way ANOVA was used to determine the relationship between infarct size and contractility (PRSW). A value of p<0.05 was considered significant.

**Results:**

Left ventricular contractility is reduced in ob/ob compared with WT and even further reduced in DKO. ACE-I improved contractility in ob/ob and DKO mice. After ischemia/reperfusion without preconditioning, infarct size was larger in DKO and ob/ob versus WT. Hypoxic preconditioning induced a strong protection in WT and a partial protection in ob/ob mice. The preconditioning potential was lost in DKO. Twelve weeks of food restriction or ACE-I restored the preconditioning potential in DKO and improved it in ob/ob.

**Conclusion:**

Delayed preconditioning is restored by food restriction and ACE-I in case of type II diabetes and the metabolic syndrome.

## Background

Tissues and organs can be protected from ensuing longer ischemia-reperfusion (IR) periods by short preceding episodes of ‘preconditioning’ ischemia [1]. However, after 26 years of research and the consistent and significant protection of cardiac preconditioning found in most experimental studies, none of these results has been translated into clinical therapies [1-5]. There are several reasons. The stimulus to induce preconditioning, such as repeated temporary coronary occlusion or aortic cross-clamping, can be harmful itself and prolongs the procedure. It was further debated whether standard maneuvers, such as cardiopulmonary bypass during cardiac surgery, themselves precondition the patient [4]. Another important issue in this failure, is the uncertainty whether this technique can effectively salvage myocardium in patients with co-morbidities, such as type 2 diabetes (T2D) or the metabolic syndrome (MS), that are frequent in the intended patient population [1,5].

The number of patients with diabetes and the metabolic syndrome increases in Western societies and reaches epidemic proportions [6-9]. The incidence of myocardial infarction is twice that of the general population and infarct size, for a given ischemic insult, is larger [7,8]. Large-scale clinical trials showed that intensive glycemic control failed to reduce cardiovascular mortality and macrovascular incidents including myocardial infarction, in comparison with standard glucose control in diabetic patients [9]. Therefore, new or additional techniques to protect the diabetic heart against ischemic damage are eagerly awaited.

Delayed preconditioning might be an interesting technique for this, since it is capable to induce cardioprotection in a controlled way for longer periods. Most of our knowledge concerning preconditioning however has been gathered from experiments in normal healthy animal models [1]. These models are not optimal to study preconditioning for the clinical population that might benefit most of it, i.e. patients with T2D or the MS. Delayed preconditioning reduces infarct size and preserves cardiac contractility in wild type (WT)-mice after ischemia-reperfusion [10], but not in models of type I diabetes [11]. Clinical studies report that type II diabetic myocardium is resistant or has a higher threshold to classical preconditioning [2,3]. The effect of delayed preconditioning on cardiac IR in mouse models of T2D and the MS has never been investigated and is the focus of this study.

Most patients with T2D or the MS are treated [12]. These treatments might additionally influence the preconditioning potential. Despite the fact that hypocaloric diet is a standard treatment in T2D and the MS [12], its cardiovascular effects are incompletely understood. A study with obese rats showed that food restriction partially restores classical preconditioning [13]. The effects of long-term food restriction on delayed preconditioning was never investigated. Food restriction increases endothelial nitric oxide synthase (eNOS)-levels in a mouse model of the metabolic syndrome [14], NO being a determinant trigger in the delayed preconditioning pathway [1]. It is therefore conceivable that food restriction restores the delayed preconditioning potential and induces cardioprotection by this mechanism.

Angiotensin-converting enzyme inhibition (ACE-I) is also a standard therapy in the treatment of hypertension in diabetic patients [12]. It was previously shown that ACE-I is capable to augment classical [15,16] and delayed preconditioning [17,18] in WT animal models, by lowering the stimulus threshold. There are no data concerning the effect of ACE-I on the delayed preconditioning in T2D and MS models. ACE-I might however impact on the delayed preconditioning potential since it is capable to block the degradation of bradykinin into inactive peptides and to increase eNOS and nitric oxide (NO)-levels [15,16,19].

Therefore, the aim of this study is to investigate the effects of food restriction and ACE-I on delayed cardiac preconditioning in diabetic or metabolic syndrome models. We tested the hypotheses that 1° mice with T2D or the MS are resistant to hypoxic delayed preconditioning; and 2° the interventions food restriction or ACE-I restore the preconditioning potential in these mouse models.

## Methods and materials

### Animal models

Experiments were conducted in 49 C57BL/6J WT-mice, 71 leptin deficient ob/ob, and 68 double knock-out (DKO)-mice with combined leptin and LDL-receptor deficiency. The ob/ob mouse is a model for T2D, featuring abdominal obesity and insulin resistance. DKO feature many characteristics of the metabolic syndrome, i.e. obesity, dyslipidemia, hypertension, insulin resistance and impaired glucose tolerance and/or diabetes [14]. These models develop left ventricular diastolic and systolic dysfunction comparable with the diabetic cardiomyopathy seen in patients [20]. Ob/ob and C57BL/6J-mice were purchased from Jackson Laboratory (Bar Harbor, Maine, USA). DKO were generated as described previously [14,20]. IR was induced at 24 weeks of age. The investigation conforms with the Guide for the Care and Use of Laboratory Animals published by the US National Institutes of Health (NIH Publication 1996). All experimental protocols were approved by the K.U. Leuven Institutional Animal Care Commission and Ethical Committee.

### Delayed preconditioning stimulus

The experimental technique was previously described [10]. Briefly, mice were placed in a plexiglas container, in which controlled oxygen levels of 6% could be induced within 30 seconds using a mixture of nitrogen and compressed air. Five cycles of 6 minutes of 6% oxygen, interspersed with 6 minutes of room air were used. In the non-preconditioned groups, only compressed air was used without nitrogen. Afterwards, mice were placed in their regular cage with food and water ad libitum for 24 hours.

### Treatments

Food intake (Ssniff, Soest, Germany) of diet-restricted ob/ob (n=7 sham, n=9 non-preconditioned, n=8 preconditioned) and DKO (n=7 sham, n=7 non-preconditioned, n=8 preconditioned) mice was restricted to 2.5 g/day, which is the normal daily intake of lean WT-mice, between 12 and 24 weeks of age [14,21]. ACE-inhibition was obtained with captopril (10 mg/kg/day) intraperitoneally from 12 until 24 weeks of age in WT (n=6 sham, n=7 non-preconditioned, n=8 preconditioned), ob/ob (n=7 sham, n=9 non-preconditioned, n=8 preconditioned) and DKO (n=7 sham, n=9 non-preconditioned, n=8 preconditioned) [22].

Experiments in untreated WT (n=8 sham, n=11 non-preconditioned, n=9 preconditioned), ob/ob (n=6 sham, n=9 non-preconditioned, n=8 preconditioned) and DKO (n=7 sham, n=7 non-preconditioned, n=8 preconditioned) mice served as controls.

### Ischemia/reperfusion

The experimental technique was previously described [10,20]. Briefly, anesthesia was induced with urethane (1.2 g/kg) and alfa-chloralose (50 mg/kg). Mice were ventilated with room air, with rectal temperature kept at 37±0.5°C. Via left thoracotomy, the left anterior descending artery (LAD), was non-traumatically occluded, 2 mm below the tip of the left auricle for 30 min. Afterwards, a reperfusion period of 1 hour was allowed. Successful coronary occlusion and reperfusion was visually verified by observing the myocardium distal to the coronary occlusion turning pale respectively blushing. In the groups without ischemia (sham), a thoracotomy and time-matched procedure was performed.

### Outcome parameters: infarct size and in vivo left ventricular contractility

The technique was previously described [10,20]. At the end of 1 hour reperfusion, a pressure-conductance catheter (1.4-Fr, SPR-839; Millar Instruments, Houston, TX) was inserted through the right carotid artery into the left ventricle. Baseline pressure-volume (PV) loops were recorded (Powerlab/ADInstruments, Castle Hill, Australia). Parallel volume and specific blood conductance were determined [10,20,21]. The inferior caval vein was compressed to obtain left ventricular PV-loops under varying loading conditions. Heart rate, systolic and end-diastolic pressure were measured. Stroke volume was determined as the difference in end-diastolic and end-systolic volume. Stroke work (SW) is the mechanical energy which the heart develops during the cardiac cycle and is calculated as the area enclosed by the PV-loop.

Preload recruitable stroke work (PRSW) is the slope of the relationship between end-diastolic volume and SW performed by the ventricle. PRSW is the most reliable and useful parameter for general contractility since it is chamber size independent and robust [23]. The slope of the end-systolic pressure-volume relationship, end-systolic elastance (E_es_), reflects left ventricular chamber end-systolic stiffness and is used as an index of contractility. Tau is the time constant of left ventricular relaxation during isovolumetric diastole. The end-diastolic PV-relationship (EDPVR) represents the compliance of the ventricular myocardium at the end of the diastole. Augmented stiffness of the ventricular wall increases the slope of the EDPVR. Arterial elastance (E_a_), a measure for afterload, is defined as the end-systolic pressure to stroke volume ratio [23].

Before excision of the heart, Evans blue (0.8 ml, 1% solution) was injected intravenously after re-occlusion of the LAD, to determine the left ventricular perfusion area at risk. The heart was cut in 1 mm-slices and the slices were stained with triphenyl-tetrazolium-chloride solution (TTC, 20 min, 1%, 37°C, pH 7.4) and fixed in paraformaldehyde (10 min, 4% solution, 20°C). All slices were weighed and photographed with a digital camera under magnification.

### Data management and statistical analysis

Analysis of the pressure-conductance data was performed using PVAN 3.2 software (Millar Instruments, Houston) as previously described [10,20,21,23].

Infarct size and area at risk were determined by the number of pixels in each zone with Adobe Photoshop 8.0 (Adobe System Inc.) multiplied by the weight of the respective slices.

All statistical analyses were performed using statistical software (Statistica 7.1, StatSoft, Tulsa, USA). Data are expressed as mean ± standard deviation. Differences between groups were analyzed for statistical significance by student’s *t*-test or one-way ANOVA followed by a Fisher’s LSD post hoc test. Factorial ANOVA was used to determine the interaction term between preconditioning and treatments, followed by a Fisher’s LSD post hoc test. Two-way ANOVA was used to determine the relationship between infarct size and contractility (PRSW). A value of p<0.05 was considered significant.

## Results

### Effect of preconditioning in untreated mice

Preconditioning without subsequent ischemia, was investigated in WT (n=8 sham preconditioned; n=8 sham non-preconditioned) and DKO (n=7 sham preconditioned; n=7 sham non-preconditioned) mice. As expected, this had no effect on the contractility parameters. These results can be found in Additional file 1.

**Figure 1:**
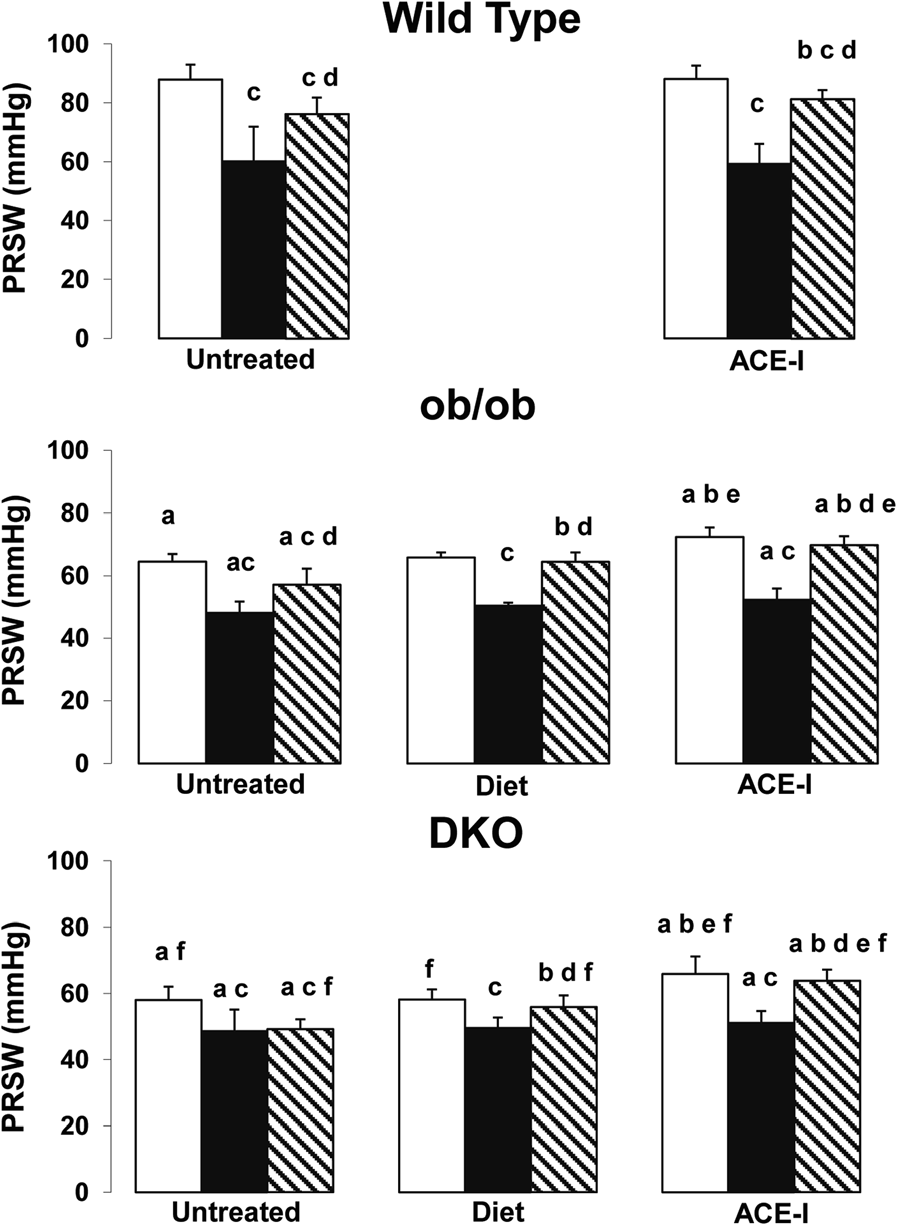
**Preload recruitable stroke work in wild type, ob/ob and double knock-out mice.** White bar: Non ischemic (sham); Black bar: Non-preconditioned and IR injury; Dashed bar: Preconditioned and IR injury^“a”^ p<0.05 versus WT same treatment, same condition (sham; non-preconditioned; preconditioned); ^“b”^ p<0.05 versus same untreated genotype, same condition; ^“c”^ p<0.05 versus sham, same genotype, same treatment; ^“d”^ p<0.05 versus non-preconditioned, same genotype, same treatment; ^“e”^ p<0.05 versus diet, same genotype, same condition; ^“f”^ p<0.05 versus ob/ob same treatment, same condition.

**Figure 2:**
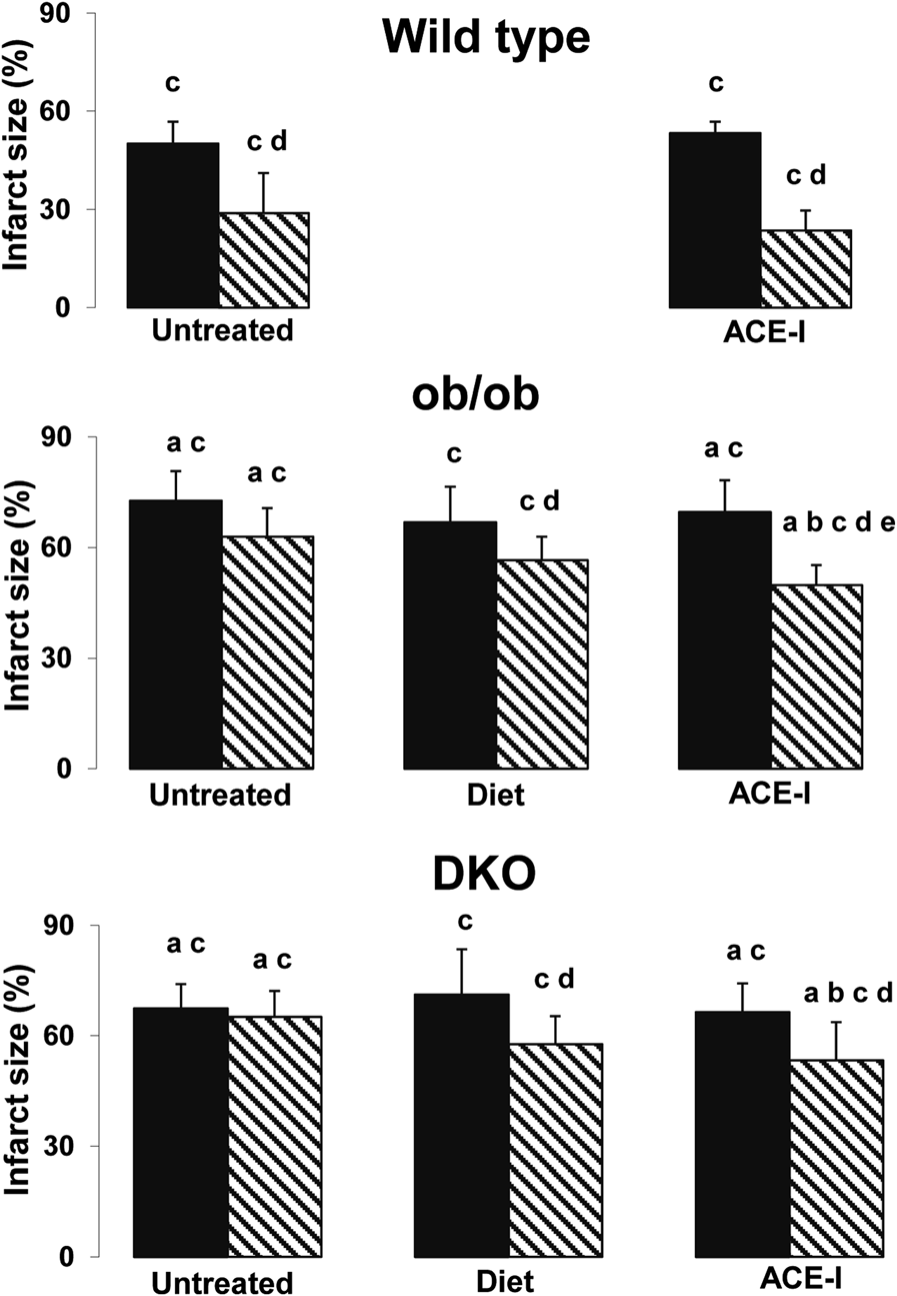
**Infarct size as % of risk zone in wild type, ob/ob and double knock-out mice.** Black bar: Non-preconditioned and IR injury; Dashed bar: Preconditioned and IR injury ^“a”^ p<0.05 versus WT same treatment, same condition (sham; non-preconditioned; preconditioned); ^“b”^ p<0.05 versus same untreated genotype, same condition; ^“c”^ p<0.05 versus sham, same genotype, same treatment; ^“d”^ p<0.05 versus non preconditioned, same genotype, same treatment; ^“e”^ p<0.05 versus diet, same genotype, same condition.

Left ventricular contractility, expressed as PRSW (Figure 1), is reduced in ob/ob compared with WT and even further reduced in DKO (Table 1). The area at risk after coronary occlusion is comparable in all groups undergoing IR. After IR without preconditioning, infarct size was larger in DKO (Table 2) and ob/ob versus WT (Figure 2). IR impaired PRSW significantly in all groups, but to a significantly lower level in DKO and ob/ob than in WT (Table 1).

**Table 1:** Load-independent hemodynamic parameters

		**Wild type**			**Ob/ob**			**DKO**		
	***Condition***	***Sham***	***NP***	***P***	***Sham***	***NP***	***P***	***Sham***	***NP***	***P***
**PRSW (mmHg)**	Untreated	87.9±5.0	60.2±11.8^c^	76.3±5.4 ^cd^	64.5±2.5 ^a^	48.1±3.6 ^ac^	57.1±5.2 ^acd^	58.0±4.1 ^af^	48.6±6.5 ^ac^	49.2±3.0 ^acf^
	Diet				65.8±1.7	50.4±1.0 ^c^	64.4±3.1 ^bd^	58.2±3.0 ^f^	49.5±3.2 ^c^	55.9±3.5 ^bdf^
	ACE-I	88.0±4.6	59.3±6.8 ^c^	81.2±3.1 ^bcd^	72.4±3.1 ^abe^	52.3±3.6 ^ac^	69.8±2.9 ^abde^	65.9±5.3 ^abef^	51.1±3.6 ^ac^	63.9±3.3 ^abdef^
**Ees (mmHg/μl)**	Untreated	8.3±2.5	6.4±1.5	8.5±1.7 ^d^	5.0±0.4 ^a^	4.8±1.7	5.6±1.2 ^a^	6.0±2.2	4.5±0.8 ^a^	4.8±2.3 ^a^
	Diet				6.3±2.2	4.4±1.2	7.3±2.2^d^	5.1±1.3	5.4±1.2	5.6±1.1
	ACE-I	6.0±1.1	6.2±1.6	8.7±1.7 ^cd^	5.3±1.7	4.2±1.3	6.4±1.6 ^ad^	5.6±1.4	4.3±1.4	5.2±3.0 ^a^
**EDPVR (mmHg/μl)**	Untreated	0.2±0.2	0.4±0.2	0.3±0.2	0.4±0.3	0.4±0.3	0.5±0.3 ^a^	0.5±0.4	0.3±0.1	0.3±0.2
	Diet				0.3±0.2	0.2±0.1	0.2±0.1 ^b^	0.3±0.1	0.2±0.1 ^b^	0.3±0.2
	ACE-I	0.2±0.2	0.4±0.4	0.2±0.1	0.4±0.1	0.3±0.1	0.3±0.1 ^bc^	0.3±0.1 ^f^	0.3±0.1	0.2±0.1

ACE-I: angiotensin-converting enzyme inhibition; DKO: double knock-out (ob/ob; LDLR-/-); EDPVR: end-diastolic pressure-volume relationship; E_es_: end-systolic elastance; IR: ischemia/reperfusion; NP: Non-Preconditioned and ischemia/reperfusion; P: Preconditioned and ischemia/reperfusion; PRSW: preload recruitable stroke work; sham: group without ischemia/reperfusion. ^“a”^ p<0.05 versus WT same treatment, same condition (sham; non-preconditioned; preconditioned); ^“b”^ p<0.05 versus same untreated genotype, same condition; ^“c”^ p<0.05 versus sham, same genotype, same treatment; ^“d”^ p<0.05 versus non-preconditioned, same genotype, same treatment; ^“e”^ p<0.05 versus diet, same genotype, same condition; ^“f”^ p<0.05 versus ob/ob same treatment, same condition.

**Table 2:** Numbers and infarct size

		**Wild type**			**Ob/ob**			**DKO**		
	***Condition***	***Sham***	***NP***	***P***	***Sham***	***NP***	***P***	***Sham***	***NP***	***P***
**N experiments (survivors)**	Untreated	8 (8)	11 (9)	9 (9)	6 (6)	9 (6)	8 (7)	7 (6)	7 (6)	8 (7)
	Diet				7 (6)	9 (6)	8 (7)	7 (6)	7 (7)	8 (8)
	ACE-I	6 (6)	7 (6)	8 (8)	7 (7)	9 (7)	8 (7)	7 (7)	9 (7)	8 (7)
**Weight (g)**	Untreated	28.3±4.6			62.4±3.8 ^a^			59.1±9.5 ^a^		
	Diet				38.3±4.7 ^b^			37.9±3.4 ^b^		
	ACE-I	27.1±3.4			62.9±9.4 ^ae^			63.6±8.5 ^ae^		
**Area at risk (% of heart)**	Untreated	0	15.4±7.6 ^c^	16.6±6.9 ^c^	0	15.6±6.1^c^	12.4±5.5 ^c^	0	18.8±7.6 ^c^	12.9±3.5 ^c^
	Diet				0	18.7±2.6 ^c^	19.4±6.6 ^c^	0	17.5±8.7 ^c^	17.9±10.7 ^c^
	ACE-I	0	19.0±5.8 ^c^	18.8±7.4 ^c^	0	16.4±3.0 ^c^	17.5±8.8 ^c^	0	14.4±5.3 ^c^	16.1±7.1 ^c^
**Infarct size (% of area at risk)**	Untreated	0	50.2±6.5 ^c^	28.9±12.3 ^cd^	0	72.7±8.0 ^ac^	62.9±7.8 ^ac^	0	67.4±6.6 ^ac^	65.1±7.1 ^ac^
	Diet				0	66.8±9.7 ^c^	56.5±6.4 ^cd^	0	71.2±12.3 ^c^	57.7±7.6 ^cd^
	ACE-I	0	53.2±3.5 ^c^	23.6±6.1 ^cd^	0	69.6±8.6 ^ac^	49.7±5.5 ^abcde^	0	66.4±7.8 ^ac^	53.3±10.4 ^abcd^

ACE-I: angiotensin-converting enzyme inhibition; DKO: double knock-out (ob/ob; LDLR-/-); NP: ischemia/reperfusion and Non-Preconditioned; P: ischemia/reperfusion and Preconditioned. ^“a”^ p<0.05 versus WT same treatment, same condition (sham; non-preconditioned; preconditioned); ^“b”^ p<0.05 versus same untreated genotype, same condition; ^“c”^ p<0.05 versus sham, same genotype, same treatment; ^“d”^ p<0.05 versus non-preconditioned, same genotype, same treatment; ^“e”^ p<0.05 versus diet, same genotype, same condition.

Preconditioning induced a strong protection in WT mice. Infarct size, stroke volume, E_es_, E_a_ and PRSW were significantly better preserved after preconditioning (Table 1, 2 and Additional file 2). In ob/ob, delayed preconditioning failed to significantly reduce infarct size (p = 0.06). Nevertheless, load-independent left ventricular contractility, as measured by PRSW, was better preserved after preconditioning. The preconditioning potential was lost in DKO mice: infarct size was not reduced and none of the contractility parameters were significantly better preserved after preconditioning. The relationship between infarct limitation and improved contractility was significantly different in DKO and WT mice. In DKO, preconditioning induced a proportional smaller improved PRSW per reduced infarct area versus WT mice (Additional file 3).

### Effect of food restriction on preconditioning

Food restriction did not improve the reduced PRSW in sham ob/ob and DKO mice (Figure 1). Food restriction did not influence infarct size and PRSW in non-preconditioned DKO and ob/ob versus the untreated groups (Table 1 and 2) [10].

After food restriction, delayed preconditioning was restored in ob/ob mice, as seen by the significantly reduced infarct size, and significantly better preserved E_es_ and PRSW. These load-independent contractility parameters reached values comparable to the non-ischemic sham group.

Furthermore, food restriction restored the preconditioning potential in DKO mice: infarct size and PRSW were significantly better preserved versus the non-preconditioned group. Left ventricular contractility, expressed as PRSW, remained worse versus food restricted and preconditioned ob/ob mice.

### Effect of ACE-I on preconditioning

ACE-I improved contractility in sham ob/ob and DKO mice, not in WT. Furthermore, PRSW was significantly better after 12 weeks of ACE-I in ob/ob and DKO versus diet (Figure 1). Without preconditioning, ACE-I did not reduce infarct size and the impact of IR injury on PRSW in any genotype versus the untreated groups (Table 1 and 2) [10].

After ACE-I treatment, the protective effect of preconditioning on PRSW and SW was increased in WT-mice versus the untreated group (Table 1 and Additional file 2).

After ACE-I treatment, delayed preconditioning was fully restored in ob/ob mice concerning stroke work, Tau, E_es_ and PRSW. Infarct size after preconditioning was even smaller than the already beneficial effect that could be obtained with diet (Figure 2).

Also in DKO mice, ACE-I treatment restored delayed preconditioning. Infarct size and PRSW were significantly better preserved than the non-preconditioned group. After ACE-I treatment and preconditioning in DKO mice, PRSW was better preserved versus food restricted preconditioned DKO and reached the level of the sham group without ischemia. After ACE-I and preconditioning, the proportional effect on infarct limitation and improved contractility was restored to the WT-level (Additional file 3).

## Discussion

Hypoxic preconditioning was capable to induce a strong protection in WT mice, but not in the DKO model of MS. Furthermore, the relationship between infarct limitation and improved contractility after preconditioning was different in DKO than in WT mice. In DKO, preconditioning induced a proportionally smaller improved PRSW per reduced infarct area versus WT mice. In ob/ob, preconditioning induced only a partial preservation of left ventricular contractility. The degree of protection was inferior to that in WT.

Since we have not directly investigated the mechanisms underlying the reduced or abolished delayed preconditioning potential, we can only hypothesize on them. This should further be investigated in depth to allow specific, additional therapeutic interventions. Potential underlying mechanisms are numerous.

First, our models are known with raised concentrations of non-esterified fatty acids, insulin resistance and hyperglycemia [14,20,21]. These metabolic anomalies induce atypical isoforms of protein-kinase C [24], which is crucial in the pathway of delayed preconditioning [1].

Second, insulin induces a preconditioning effect [5]. It was shown that at 24 weeks, both ob/ob and DKO mice have increased insulin levels [14,20]. It is therefore possible that mouse models with hyperinsulinemia are in a permanent preconditioned state or require a higher threshold to preconditioning [5,25].

Third, previous studies demonstrated the importance of mitochondrial reactive oxygen species (ROS), generated in response to a preconditioning stimulus [1,2]. Increased oxidative stress is demonstrated in our mouse models [14,20]. It is therefore possible that increased ROS-levels induce a permanent preconditioned state or induce a higher threshold to preconditioning.

Fourth, the role of NO in IR injury and preconditioning is complex and depends on its concentration and cellular origin [1,26]. eNOS is a trigger in the delayed preconditioning pathway. Endothelial dysfunction and reduced nitric oxide (NO) bioavailability is present in T2D and the MS [27]. During IR injury, iNOS has a beneficial role in normal myocardium since iNOS knock-out non-diabetic mice show a larger infarct size versus WT-controls [28]. The small magnitude of increase in iNOS levels in this situation is important, because the up regulation of iNOS has a pronounced dose-dependent effect: beneficial at low levels but toxic at high levels [1,26]. In diabetic myocardium, basal iNOS-levels are 3 times higher than in non-diabetic myocardium [29]. Furthermore, the increase of iNOS-levels in ischemic areas after IR injury is 2.6 times larger than in the ischemic areas of non-diabetic myocardium [29]. The detrimental role of these high iNOS-levels during IR injury in diabetic myocardium was confirmed in another study [28], in which iNOS knock-out diabetic mice showed a smaller infarct size and reduced caspase-3 activity versus control diabetic mice. This might explain the increased sensitivity of our diabetic mice to IR injury. In the delayed preconditioning pathway, iNOS has a critical beneficial role [1]. Targeted deletion of the iNOS-gene abrogates delayed preconditioning, suggesting that iNOS is a common effector of cardioprotection [26]. Since basal iNOS levels are higher, it is conceivable that diabetic mice have a disturbed threshold or are even already in a permanent maximally protected or preconditioned state.

Fifth, delayed preconditioning is regulated through an up regulation of several other cardioprotective proteins [1]. Previous studies focused mainly on the gene expression profile after delayed preconditioning in healthy subjects [30] and the effect of diabetes or the metabolic syndrome on this has never been investigated.

Finally, although a plethora of mechanisms are involved in preconditioning, it is widely accepted that all these mechanisms converge at the inhibition of the mitochondrial permeability transition pore (mPTP) opening at early reperfusion [31]. Inhibiting mPTP opening will prevent the cardiomyocytes to undergo cellular necrosis and apoptosis. No data are available about mPTP-inhibition in our mouse models of T2D and the MS.

In an attempt to restore delayed preconditioning in ob/ob and DKO mice, therapeutic strategies were investigated. After food restriction or ACE-I, hypoxic preconditioning reduced infarct size and preserved PRSW up to the level without ischemia in both ob/ob and DKO. Furthermore, ACE-I restored the proportional effect of preconditioning on infarct limitation and contractility improvement to the WT-level. Comparable with our previous study [21], we found that diet or ACE-I without preconditioning did not reduce the impact of IR injury in these mice models, in contrast with the regained protection by delayed preconditioning potential in this study. The regained preconditioning potential is thus independent from the direct effects of ACE-I on IR injury. A possible explanation is that food restriction and ACE-I are not capable to influence insulin levels, glycemia, cholesterol, ROS, eNOS and iNOS sufficiently to see an effect on IR injury without previous preconditioning. Nevertheless, these parameters might have been restored sufficiently to induce again a threshold for delayed preconditioning.

It is already shown that food restriction reduces insulin levels and ACE-I improves insulin sensitivity [14,19]. Furthermore, ACE-I reduces glycemia in ob/ob and DKO mice and food restriction lowers cholesterol levels in ob/ob mice [21]. This can have lowered the threshold to delayed preconditioning since insulin has a preconditioning effect [5,25], and hyperglycemia and hypercholesterolemia are key player in the induction of atypical isoforms of protein-kinase C [24], which is crucial in the delayed preconditioning pathway [1]. In the aging heart, an impaired protein-kinase C translocation is one of the reasons responsible for the impaired preconditioning potential [32].

Food restriction partially restores classical preconditioning in senescent animals, but in combination with exercise, this restoration becomes complete. A restored norepinephrine release was suggested as the underlying mechanism [32]. Furthermore, food restriction induces up-regulation of eNOS in DKO mice [14]. ACE-I is capable to block the degradation of bradykinin into inactive peptides and to increase eNOS-levels [15,16,19,33]. eNOS is a trigger in the delayed preconditioning pathway [1], which might explain the regained preconditioning potential.

Food restriction prevents ROS production [34] and also ACE-I has well known anti-oxidative activity [15,16,19]. Since an increase in ROS formation is an important trigger of delayed preconditioning [1], it is possible that these treatments reduce the increased baseline ROS-level in diabetic myocardium and restore the threshold for delayed preconditioning [1].

Another possible explanation why the treatments induced cardioprotection after preconditioning but did not have a direct effect during IR injury, is the different role and levels of iNOS in both pathways. As described earlier, basal iNOS-levels are higher and the increase of iNOS-levels after IR injury is larger than in non-diabetic myocardium [29] with a deleterious role in diabetic myocardium during IR injury [28]. In contrast with this, a small increase in iNOS-levels is critical to induce its effects as common end-effector in the delayed preconditioning pathway [1]. No studies were conducted to study the effect of food restriction on iNOS-levels in myocardium, but it was shown in a normotensive rat model that ACE-I reduces iNOS-levels with 24% [33]. It is tempting to speculate that ACE-I did not reduce iNOS-levels sufficiently to reduce its toxic high-dose level during IR injury but enough to restore the threshold to induce delayed precondition (by a small iNOS increase). Further investigations need to be performed to elucidate the role of these underlying mechanisms.

## Conclusion

Hypoxic preconditioning induces a strong protection in WT and a partial protection in ob/ob mice. The preconditioning potential is lost in DKO. Twelve weeks of food restriction or ACE-I restores the preconditioning potential in DKO and improves it in ob/ob. After preconditioning, PRSW is preserved to the level without ischemia, after 12 weeks food restriction or ACE-I. As previously shown, food restriction and ACE-I did not protect the diabetic myocardium against IR injury. The regained preconditioning potential appears to be independent from the direct effects of food restriction ACE-I on IR injury.

## Abbreviations

ACE: Angiotensin-converting enzyme;ACE-I: Angiotensin-converting enzyme inhibition;DKO: Double knock-out;Ea: Arterial elastance;EDPVR: End-diastolic pressure volume relationship;Ees: End-systolic elastance;eNOS: Endothelial nitric oxide synthase;iNOS: Inducible nitric oxide synthase;IR: Ischemia-reperfusion;LAD: Left anterior descending artery;LDL: Low density lipoprotein;LDLR: Low density lipoprotein receptor;mPTP: Mitochondrial permeability transition pore;NO: Nitric oxide;NP: Non-Preconditioned and ischemia/reperfusion;Ob/ob: Leptin deficient mouse model for type II diabetes;P: Preconditioned and ischemia/reperfusion;Ped: End-diastolic pressure;PRSW: Preload recruitable stroke work;Psys: Systolic pressure;PV: Pressure-volume;ROS: Reactive oxygen species;STAT-3: Signal transducer and activator of transcription-3;SW: Stroke work;TTC: Triphenyl-tetrazolium chloride;WT: Wild type

## Competing interest

The authors declare that they have no competing interest.

## Authors’ contributions

GVDM carried out the mice breeding, treatments, preconditioning, PV-loop experiments and infarct size determination, data and statistical analysis, and drafted the manuscript. IN, AV and WO contributed to the mice breeding, treatments and preconditioning. WF participated in the design of the study and general supervision. PH designed the study, obtained funding, did supervision of the analysis and interpretation of data, and revised the manuscript for important intellectual content. All authors have read and approved the final manuscript.

## Supplementary Material

Additional file 1 Hemodynamic parameters and infarct size of the non-preconditioned and preconditioned wild type and DKO, without subsequent ischemia (shams). Click here for file

Additional file 2 Hemodynamic parameters. Click here for file

Additional file 3 Relationship between infarct limitation and improved contractility. Click here for file
