# Supplementary material for: Angiotensin-converting enzyme inhibition and food restriction restore delayed preconditioning in diabetic mice
Source: Cardiovasc Diabetol. 2013 Feb 23;12:36. doi: 10.1186/1475-2840-12-36 (PMC3598767; doi:10.1186/1475-2840-12-36)
Supplement: Additional file 1 — Hemodynamic parameters and infarct size of the non-preconditioned and preconditioned wild type and DKO, without subsequent ischemia (shams). [file 1475-2840-12-36-S1.docx]

|  | **Wild Type** | | **DKO** | |
| --- | --- | --- | --- | --- |
|  | **Sham non preconditioned** | **Sham preconditioned** | **Sham non preconditioned** | **Sham preconditioned** |
| **Numbers (survivors)** | 8 (8) | 8 (8) | 7(6) | 7 (7) |
| **Area at risk (% of heart)** | 0 | 0 | 0 | 0 |
| **Infarct size (% of area at risk)** | 0 | 0 | 0 | 0 |
| **Heart rate (bpm)** | 570±40 | 549±51 | 525±31 | 522±48 |
| **Stroke volume (µl)** | 16.0±5.6 | 18.8±6.9 | 10.9±5.2 | 14.2±6 |
| **Stroke work (mmHg*µl)** | 1197±315 | 1206±658 | 656±269 | 852±494 |
| **Psys (mmHg)** | 75.6±11.6 | 73.4±13.5 | 65.6±4.9 | 70.1±8.5 |
| **Ped (mmHg)** | 2.9±0.7 | 4.2±0.9 ^a^ | 4.1±1.1 | 3.7±1.5 |
| **PRSW (mmHg)** | 87.9±5.0 | 86±5.6 | 58.0±4.1 | 57.5±2.1 |
| **Ees (mmHg/µl)** | 8.3±2.5 | 7.6±1.7 | 6.0±2.2 | 4.9±1 |
| **Tau (ms)** | 6.5±1.2 | 6.4±1.6 | 6.8±0.9 | 6.2±0.7 |
| **EDPVR (mmHg/µl)** | 0.2±0.2 | 0.5±0.5 | 0.5±0.4 | 0.4±0.3 |
| **Ea (mmHg/µl)** | 4.6±1.5 | 3.7±0.6 | 5.8±2.6 | 4.4±1.2 |

**Additional file 1: Hemodynamic parameters and infarct size of the non-preconditioned and preconditioned wild type and DKO, without subsequent ischemia (shams)**

DKO: double knock-out (ob/ob; LDLR-/-); ^“a”^ p<0.05 versus non preconditioned sham group, same genotype
