# Supplementary material for: Angiotensin-converting enzyme inhibition and food restriction restore delayed preconditioning in diabetic mice
Source: Cardiovasc Diabetol. 2013 Feb 23;12:36. doi: 10.1186/1475-2840-12-36 (PMC3598767; doi:10.1186/1475-2840-12-36)
Supplement: Additional file 2 — Hemodynamic parameters. [file 1475-2840-12-36-S2.doc]

**Additional file 2**: Hemodynamic parameters

|  |  | **Wild Type** | | | **Ob/ob** | | | **DKO** | | |
| --- | --- | --- | --- | --- | --- | --- | --- | --- | --- | --- |
|  | ***Condition*** | ***Sham*** | ***NP*** | ***P*** | ***Sham*** | ***NP*** | ***P*** | ***Sham*** | ***NP*** | ***P*** |
| **Heart rate (bpm)** | Untreated | 570±40 | 567±61 | 561±49 | 532±45 | 552±31 | 542±59 | 525±31 | 505±35 ^f^ | 496±18 ^a^ |
|  | Diet |  |  |  | 511±26 | 494±32 | 516±40 | 503±34 | 519±54 | 536±32 ^b^ |
|  | ACE-I | 546±36 | 518±36 | 547±77 | 503±35 | 493±38 | 529±45 | 512±37 | 501±70 | 528±69 |
| **Stroke volume (µl)** | Untreated | 16.0±5.6 | 6.9±3.3 ^c^ | 10.2±2.7 ^cd^ | 15.4±3.0 | 12.4±4.7 ^a^ | 15.5±5.9 ^a^ | 10.9±5.2 | 10.4±3.9 | 10.2±4.7 |
|  | Diet |  |  |  | 14.2±6.3 | 14.6±7.5 | 13.9±6.6 | 12.1±5.5 | 11.4±5.1 | 10.0±3.0 |
|  | ACE-I | 17.5±4.8 | 7.9±1.6 ^c^ | 13.0±3.4 ^d^ | 14.2±3.4 | 13.3±4 ^a^ | 16.5±2.8 | 15.3±6.5 | 12.5±3.2 ^a^ | 12.8±4.8 |
| **Stroke work (mmHg*µl)** | Untreated | 1197±315 | 277±171 ^c^ | 417±107 ^c^ | 861±192 ^a^ | 524±206 ^ac^ | 732±282 ^a^ | 656±269 ^a^ | 458±178 | 469±230 |
|  | Diet |  |  |  | 857±578 | 689±362 | 735±364 | 668±273 | 617±306 | 573±229 |
|  | ACE-I | 963±378 | 359±175 ^c^ | 670±23 ^bd^ | 798±269 | 622±216 ^a^ | 940 ± 302 ^d^ | 781±441 | 566±183 | 659±298 |
| **Psys (mmHg)** | Untreated | 75.6±11.6 | 56.8±9.6 ^c^ | 58.9±12 ^c^ | 68.2±8.1 | 62.9±2.8 | 61.5±3.1 | 65.6±4.9 | 65.6±8.1 | 58.2±3.5 ^c^ |
|  | Diet |  |  |  | 69.7±11.3 | 60.5±6.5 | 69.0±8.3 ^b^ | 70.0±11 | 65.5±5.7 | 74.9±19.6 |
|  | ACE-I | 65.5±11.2 | 60.5±21.9 | 64.1±6.7 | 66.7±6.4 | 59.4±8.0 | 67.1±11.0 | 60.6±6.2 | 56.3±8.7 ^e^ | 63.2±2.7 ^b^ |
| **Ped (mmHg)** | Untreated | 2.9±0.7 | 3.0±1.2 | 2.5±0.5 | 3.6±0.7 | 6.6±3.6 ^a^ | 6.6±0.9 ^ac^ | 4.1±1.1 ^a^ | 7.2±2.1 ^ac^ | 5.6±3.5 ^a^ |
|  | Diet |  |  |  | 3.1±1.1 | 4.5±1.2 | 4.3±1.5 ^b^ | 3.8±1.4 | 4.4±1.6 ^b^ | 4.9±2.3 |
|  | ACE-I | 3.6±2.2 | 3.1±2.5 | 3.7±1.0 ^b^ | 4.5±1.0 ^e^ | 5.0±1.6 | 5.0±2.0 | 3.6±1.0 | 3.7±1.4 ^be^ | 3.9±1.2 |
| **Tau (ms)** | Untreated | 6.5±1.2 | 7.2±1.9 | 6.0±0.8 | 6.1±0.5 | 6.2±0.8 | 6.8±0.6 | 6.8±0.9 | 7.3±0.8 ^f^ | 7.4±0.6 ^a^ |
|  | Diet |  |  |  | 6.9±0.6 ^b^ | 7.7±0.8 ^b^ | 6.9±1.4 | 6.7±0.6 | 7.3±1.3 | 7.6±0.9 |
|  | ACE-I | 6.8±1.6 | 7.6±1.6 | 5.9±0.9 ^d^ | 7.9±1.2 ^b^ | 8.8±1.6 ^b^ | 6.4±1.0 ^cd^ | 7.3±0.7 | 7.1±1.1 ^f^ | 7.2±1.4 ^a^ |
| **Ea (mmHg/µl)** | Untreated | 4.6±1.5 | 8.4±2.5 ^c^ | 5.7±2.3 ^d^ | 3.8±0.7 | 5.0±2.2 ^a^ | 3.6±1.1 ^a^ | 5.8±2.6 | 5.3±1.7 ^a^ | 5.3±2.3 |
|  | Diet |  |  |  | 4.8±1.9 | 4.8±2.9 | 5.2±2.0 | 5.4±2.4 | 5.8±1.9 | 7.5±3.8 |
|  | ACE-I | 3.2±0.7 | 6.9±1.4 ^c^ | 4.4±0.7 ^cd^ | 4.3±1.5 | 4.1±1 ^a^ | 3.3±1.1 ^ae^ | 3.3±1.4 ^b^ | 3.8±1.1 ^ae^ | 4.7±1.6 |

ACE-I: angiotensin-converting enzyme inhibition; DKO: double knock-out (ob/ob; LDLR-/-); E_a_: arterial elastance; IR: ischemia/reperfusion; NP: Non Preconditioned and ischemia/reperfusion; P: Preconditioned and ischemia/reperfusion; P_ed_: end-diastolic pressure; P_sys_: systolic pressure; sham: group without ischemia/reperfusion. ^“a”^ p<0.05 versus WT same treatment, same condition (sham-non preconditioned-preconditioned); ^“b”^ p<0.05 versus same untreated genotype, same condition; ^“c”^ p<0.05 versus sham, same genotype, same treatment; ^“d”^ p<0.05 versus non preconditioned, same genotype, same treatment; ^“e”^ p<0.05 versus diet, same genotype, same condition; ^“f”^ p<0.05 versus ob/ob same treatment, same condition
