# Supplementary material for: Angiotensin-converting enzyme inhibition and food restriction restore delayed preconditioning in diabetic mice
Source: Cardiovasc Diabetol. 2013 Feb 23;12:36. doi: 10.1186/1475-2840-12-36 (PMC3598767; doi:10.1186/1475-2840-12-36)
Supplement: Additional file 3 — Relationship between infarct limitation and improved contractility. [file 1475-2840-12-36-S3.doc]

**Additional file 3: Relationship between infarct limitation and improved contractility**


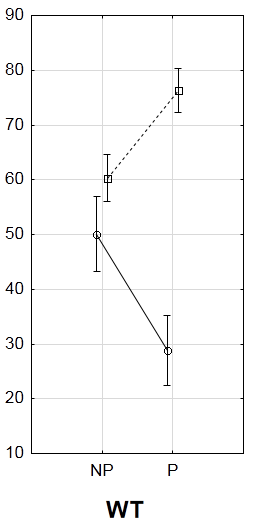

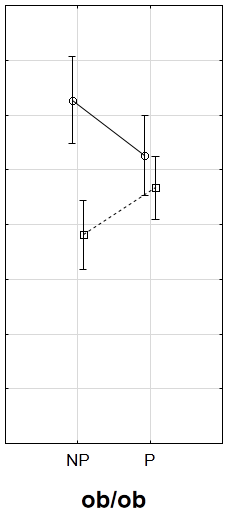

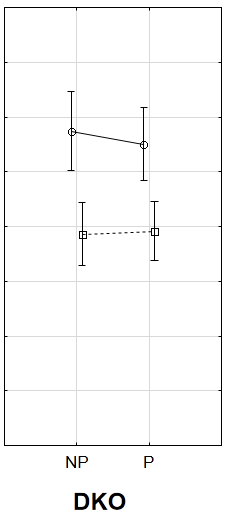


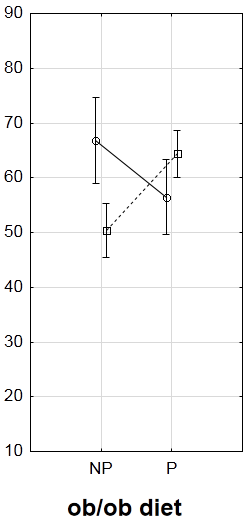

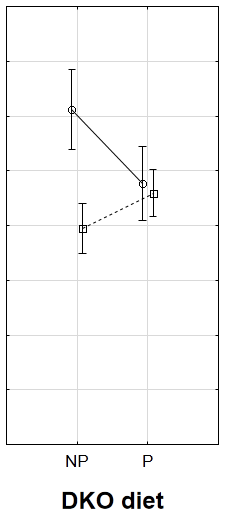


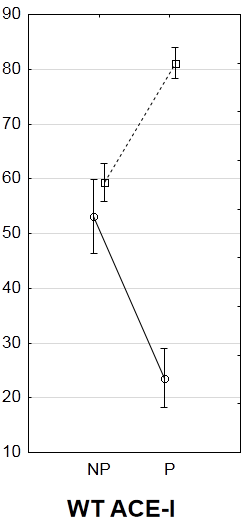

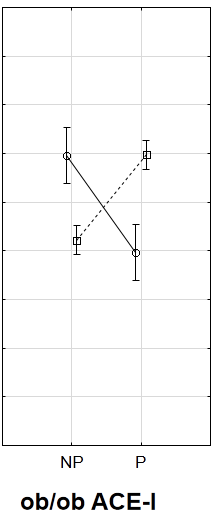

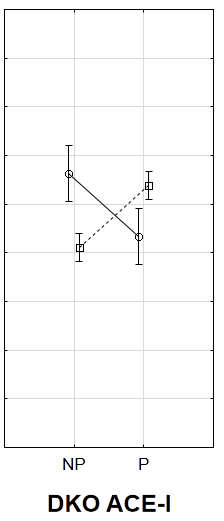


Solid lines: Infarct of risk area (%); Dotted lines: PRSW

ACE-I: angiotensin-converting enzyme inhibition; DKO: double knock-out (ob/ob; LDLR-/-); NP: non preconditioned; P: preconditioned; WT: wild type

The relationship between infarct limitation and improved contractility was significantly different in DKO and WT mice, but not in ob/ob. In DKO, preconditioning induced a proportional smaller improved PRSW per reduced infarct area versus WT mice. An infarct size reduction of 1%, corresponds with an increased PRSW of 1.25% in WT and only 0.53% in DKO. After diet and preconditioning in DKO, this relationship was partially restored (increase PRSW of 0.95% per 1% reduced infarct size, p=0.1 versus untreated DKO) but significantly after ACE-I (increase PRSW of 1.91% per 1% reduced infarct size, p=0.003 versus untreated DKO).
